# Polygenic risk of social isolation behavior and its influence on psychopathology and personality

**DOI:** 10.1038/s41380-024-02617-2

**Published:** 2024-05-30

**Authors:** Adam J. Socrates, Niamh Mullins, Ruben C. Gur, Raquel E. Gur, Eli Stahl, Paul F. O’Reilly, Abraham Reichenberg, Hannah Jones, Stanley Zammit, Eva Velthorst

**Affiliations:** 1 Department of Psychiatry, Icahn School of Medicine at Mount Sinai, One Gustave L. Levy Pl., New York, NY 10029 USA; 2 Department of Genetics and Genomic Sciences, Icahn School of Medicine at Mount Sinai, One Gustave L. Levy Pl., New York, NY 10029 USA; 3 Charles Bronfman Institute for Personalized Medicine, Icahn School of Medicine at Mount Sinai, One Gustave L. Levy Pl., New York, NY 10029 USA; 4 Department of Psychiatry, Perelman School of Medicine and the Lifespan Brain Institute, Penn Medicine and Children’s Hospital of Philadelphia, University of Pennsylvania, 3400 Spruce, Philadelphia, PA 19104 USA; 5 MRC Integrative Epidemiology Unit, University of Bristol, Bristol, BS8 2PR UK; 6 Department of Population Health Sciences, Bristol Medical School, University of Bristol, Bristol, BS8 2PR UK; 7 Centre for Academic Mental Health, Bristol Medical School, University of Bristol, Bristol, BS8 2PR UK; 8 Division of Psychological Medicine and Clinical Neurosciences, MRC Centre for Neuropsychiatric Genetics and Genomics, Cardiff University, Cardiff, CF24 4HQ UK; 9 Department of Research, Mental Health Organization “GGZ Noord-Holland-Noord,”, Heerhugowaard, The Netherlands; 10 Present Address: Regeneron Genetics Centre, Tarrytown, NY USA

**Keywords:** Autism spectrum disorders, Genetics

## Abstract

Social isolation has been linked to a range of psychiatric issues, but the behavioral component that drives it is not well understood. Here, a genome-wide associations study (GWAS) was carried out to identify genetic variants that contribute specifically to social isolation behavior (SIB) in up to 449,609 participants from the UK Biobank. 17 loci were identified at genome-wide significance, contributing to a 4% SNP-based heritability estimate. Using the SIB GWAS, polygenic risk scores (PRS) were derived in ALSPAC, an independent, developmental cohort, and used to test for association with self-reported friendship scores, comprising items related to friendship quality and quantity, at age 12 and 18 to determine whether genetic predisposition manifests during childhood development. At age 18, friendship scores were associated with the SIB PRS, demonstrating that the genetic factors can predict related social traits in late adolescence. Linkage disequilibrium (LD) score correlation using the SIB GWAS demonstrated genetic correlations with autism spectrum disorder (ASD), schizophrenia, major depressive disorder (MDD), educational attainment, extraversion, and loneliness. However, no evidence of causality was found using a conservative Mendelian randomization approach between SIB and any of the traits in either direction. Genomic Structural Equation Modeling (SEM) revealed a common factor contributing to SIB, neuroticism, loneliness, MDD, and ASD, weakly correlated with a second common factor that contributes to psychiatric and psychotic traits. Our results show that SIB contributes a small heritable component, which is associated genetically with other social traits such as friendship as well as psychiatric disorders.

## Introduction

Social contact is essential for surviving and thriving in human societies [1]. As such, having limited contact with other people, or social isolation, can have detrimental effects on both physical and mental health. There is evidence that lack of social contact is associated with schizophrenia [2, 3], autism spectrum disorder [3], and depression [4], as well as with medical conditions such as cardiovascular disease [5] and diabetes [6]. Longitudinal studies indicate that Social-isolation can predate mental health issues and have a strong causal effect on poor mental health outcomes [4, 7, 8]. These issues have been acutely brought to light in the context of the Covid-19 pandemic, in which forced social isolation has had a substantial negative effect on mental health [9]. Social isolation has been strongly associated with the development of psychosis, and it has been hypothesized that this contribution may be due to isolated individuals with negative, delusional, or paranoid thoughts not having the opportunity to apply or test these beliefs in real world situations, and therefore not being challenged and not having these delusions corrected by actual social interactions [10, 11].

Despite the impact of social isolation on mental and physical health, it remains among the least studied factors in psychiatric disorders, limiting understanding of etiology and causality with regards to psychiatric disorders [12–15]. Associations between genetics and traits related to social contact such as feelings of loneliness (feelings of distress or discomfort from being alone) and sociability (the ability to connect and socialize with others) have been noted [16]. More recently, Bralten et al. investigated the genetic underpinnings of a sociability phenotype combining behavioral and internalizing traits [17]. However, the existence and influence of an exclusive genetic predisposition towards social isolation *behaviors* specifically (SIB), i.e., action that leads to isolation, as distinct from the feelings that may potentially motivate or stem from such behavior, is yet to be established. Consequently, there is a gap in our knowledge about the extent to which SIB may represent a *causal and independent* risk for poor mental and physical health instead of being merely a direct *consequence* of other (clinical) symptomatology, for example due to stress or feelings of paranoia.

Twin studies have demonstrated that there is a similar genetic influence on both social isolation (as measured by access to social support; 40%) and loneliness (38%), but that they are only moderately genetically correlated, reflecting partially distinct constructs [18]. However, to our knowledge no prior study has carried out a genome-wide association study (GWAS) to elucidate the polygenic component of the purely behavioral aspects of SIB, as separate and distinct from feelings such as loneliness or relationship satisfaction. This information is pertinent, as the behavior itself could be partially driven by genetic factors, and could be detected and modified, providing early intervention targets if found to be on the causal pathway between inherited genetic variation and psychiatric disorders [19].

In order to better understand the genetic factors that influence SIB, the present study (1) conducted a novel GWAS for SIB in the UK Biobank cohort by meta-analyzing 4 behavioral social isolation traits; (2) derived Polygenic Risk Scores (PRS) from this GWAS for individuals in the Avon Longitudinal Study of Parents and Children (ALSPAC, UK) and used to examine associations with social traits for GWAS validation; (3) examined the genetic correlation between SIB and psychiatric disorders using GWAS results from the Psychiatric Genomics Consortium (PGC), as well as genetic correlations with personality and psychological traits such as neuroticism and loneliness; (4) Mendelian Randomization (MR) was applied to estimate causal effects between SIB and these traits/disorders; and (5) Genomic structural equation modeling (Genomic SEM) was used to model the shared genetic architecture of SIB and these traits.

## Results

### GWAS

**Fig. 1:**
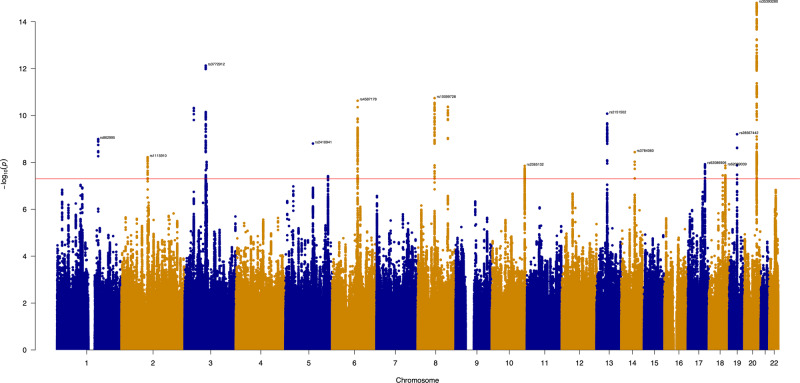
Manhattan plot for social isolation behavior after conditioning on psychiatric disorders, based on the meta-analysis of 4 traits. The red horizontal line denotes genome-wide significance (*P* < 5 × 10^−08^). See Supplementary Table 5 for details on genome-wide significant SNPs.

To investigate genetic propensity towards social isolation behavior (SIB), a GWAS was performed in the UK Biobank, based on a composite of 4 self-reported behavioral traits, constructed so higher scores represent increased SIB. A GWAS was performed on each of the 4 traits, after which Linkage disequilibrium (LD) score correlation revealed that the individual traits were genetically correlated (see Supplementary Table 4). These were meta-analyzed with Multi-Trait Analysis of GWAS (MTAG) to produce a single GWAS before being conditioned on schizophrenia, major depressive disorder (MDD), and autism spectrum disorder (ASD), using multi-trait conditional and joint analysis (mtCOJO) to remove the effect of these psychiatric conditions. The final GWAS identified 19 loci, post-conditioning 17 loci remained at genome-wide significance (*P* < 5 × 10^−08^; see Fig. 1).

The majority of the single nucleotide polymorphisms (SNPs) found to be associated with SIB were not previously associated with psychiatric or neurodevelopmental disorders. However, there are several exceptions. For example, the top lead SNP (rs67777906; *P* = 1.80 × 10^−15^) is situated in the ARFGEF2 gene, implicated in both bipolar disorder (BD) and schizophrenia but showing opposite directions of effect and hence may be a marker that could be used to differentiate between the two [20], as well as being linked to post-traumatic stress disorder (PTSD) [21, 22]. The second top SNP in chromosome 8, and the fourth top hit overall (rs2721942; 1.47 × 10^−10^), has also been associated with PTSD [23]. In chromosome 19, the lead SNP (rs28567442; *P* = 6.31 × 10^−10^) is embedded in ZNF536, implicated in the development of the forebrain, and associated with schizophrenia [23]. Other genome-wide significant SNPs are in genes associated with schizophrenia (rs6125539; 4.72 × 10^−09^; CSE1L) [24] and impulsivity (rs1248860; 9.51 × 10^−09^; CADM2) [25]. In chromosome 13, rs17057528 (*P* = 8.82 × 10^−09^) is in DIAPH3, identified as an autism risk gene [26], and is also implicated in hearing loss and impairment of speech perception [27].

### Polygenic risk scores

#### ALSPAC

To validate the SIB GWAS and PRS in an independent cohort, as well as explore its generalizability to a developmental cohort, PRS were generated in ALSPAC using the 13 significance thresholds for SNP inclusion (*P*_T_ < 5 × 10^−08^, *P*_T_ < 5 × 10^−07^, *P*_T_ < 5 × 10^−06^, *P*_T_ < 5 × 10^−05^, *P*_T_ < 5 × 10^−04^, *P*_T_ < 0.001, *P*_T_ < 0.01, *P*_T_ < 0.05, *P*_T_ < 0.1, *P*_T_ 0.2, *P*_T_ < 0.3, *P*_T_ < 0.4, *P*_T_ < 0.5; increasing in number of SNPs as the thresholds increase). The PRS were used to examine associations with friendship scores, comprising the 5 items relating to peer contact in *n* = 4934 (at age 12) and *n* = 2909 (at age 18) participants of the ALSPAC cohort (see Supplementary Tables 2 and 3 for details on friendship score items). As the genetic variants that contribute to SIB were ascertained in an adult sample, we expected the effect of the same genetic variants in a younger sample to be less pronounced, as genetically influenced behaviors may yet to be manifest.

The SIB PRS were not associated with friendship scores at age 12. At age 18, friendship score was significantly associated with the SIB PRS at the *P*_T_ = 0.05 and *P*_T_ = 0.1 threshold, with the latter being the most strongly associated (*r*^*2*^ = 0.006, *P* = 0.001*;* see Supplementary Tables 6 and 7 for full results). The fewer SNPs included, the less predictive the model in terms of p-value, with the genome-wide significant only SNPs not associated with the friendships scores. This demonstrates that there are SNPs that do not reach genome-wide significance (5 × 10^−08^) in the GWAS that still have a signal to detect SIB and contribute to the predictive power of the SIB PRS.

#### LD score correlation

LD score correlation was performed to investigate genetic correlations between SIB in the UK Biobank and schizophrenia, MDD, ASD, anorexia nervosa (AN), and BD from the Psychiatric Genomics Consortium (PGC). Three of these psychiatric disorders were correlated with SIB, with ASD having the strongest genetic correlation (*rg* = 0.23, SE = 0.048, *P* = 2.25 × 10^−06^), followed by schizophrenia (*rg* = 0.102, SE = 0.028, *P* = 0.0002) and MDD (*rg* = 0.093, SE = 0.035, *P* = 0.009). This is in line with previous research which found negative genetic correlations with these outcomes and sociability [17]. AN and BD were not significantly genetically correlated with SIB (*rg* = −0.073, SE = 0.041, *P* = 0.073; *rg* = −0.018, SE = 0.035, *P* = 0.61). The results indicate that SIB genetics are associated with the genetics of certain psychiatric disorders and may form part of the genetic basis for them. This could occur if the genetics of SIB have downstream effects on behavior that could increase risk of symptoms and eventual diagnosis, or if the diagnosis itself leads to increased SIB.

LD score correlation was performed on anxiety, extraversion, neuroticism, loneliness, and educational attainment. Loneliness (*rg* = 0.29, SE = 0.031, *P* = 2.38 × 10^−20^) was genetically correlated with SIB, suggesting the behavioral and perceptual aspects of social isolation are influenced by partially similar genetic factors. Educational attainment and extraversion were also significantly correlated with SIB (*rg* = 0.13, SE = 0.025, *P* = 2.11 × 10^−07^; *rg* = −0.44, SE = 0.07, *P* = 1.29 × 10^−11^). The latter is negatively correlated as might be expected, but the former’s positive correlation is more surprising, and may be due to the way the phenotype is measured (i.e. *years* of education). Genetic predisposition to more advanced education in the general population could potentially be linked to less social behavior due to prioritization of study and career over socializing (see Supplementary Table 8 for full results).

Using LD score correlation, the SNP-heritability of SIB after conditioning on the genetically correlated psychiatric disorders was estimated to be *h*^*2*^ = 0.04 (SE = 0.0022, *P* = 8.95 × 10^−77^), suggesting a small but significant SNP-based heritable component.

#### Mendelian randomization

Using the MR-Egger method to account for horizontal pleiotropy, and performing bi-directional MR, there was no evidence of causal relationships between SIB and any of the genetically correlated psychiatric disorders or traits in either direction (see Table 1 below). MR-Egger is particularly robust for ascertaining causality due to allowing of pleiotropic effects i.e., variants in the model can contribute to multiple traits as is likely the case with the genetics of complex behavioral traits. However, this flexibility means weak-instrument bias, a larger estimate variance, and a loss of power. The inverse-variance weighting (IVW) method has more power, but the likely presence of horizontal pleiotropy in behavioral trait genetics means causal effects are likely to be biased. See Supplementary Table 9 for full results, including inverse variance weighted (IVW) MR.

**Table 1:** Mendelian randomization results for MR-Egger analyses.

Exposure	Outcome	N SNPs	B	SE	*P*
SIB	ASD	65	−69.360	52.57	0.192
ASD	SIB	23	0.020	0.01	0.025
SIB	SCZ	65	1.008	54.72	0.985
SCZ	SIB	313	0.004	0.00	0.225
SIB	MDD	65	−70.943	52.86	0.184
MDD	SIB	34	0.000	0.01	0.974
SIB	Edu	65	0.361	0.28	0.196
Edu	SIB	440	0.085	0.05	0.120
SIB	Extra	65	−0.029	0.41	0.945
Extra	SIB	8	0.133	0.14	0.386
SIB	Loneliness	63	0.789	0.32	0.016
Loneliness	SIB	53	0.429	0.16	0.010

*N SNPS* number of SNPs in exposure, *B* Beta, *SE* standard errors, *P* *p*-value, *SIB* social isolation behavior, *ASD* autism spectrum disorder, *SCZ* schizophrenia, *MDD* major depressive disorder, *Edu* educational attainment, *Extra* extraversion.

#### Genomic SEM

To ascertain the genetic architecture, we initially tested a model in which all 11 traits (SIB, plus schizophrenia, MDD, ASD, AN, BD, anxiety, extraversion, neuroticism, loneliness, and educational attainment loaded onto a single common factor. Model fit was fairly poor (χ2 = 1756.37, AIC = 1800.37, CFI = 0.53, SRMR = 0.134), with MDD, Neuroticism, and loneliness most strongly loading onto the common factor, and educational attainment, AN and extraversion loading negatively onto this latent factor. We then removed the negatively loading traits, as well as anxiety due to it being underpowered in the model and produced a model in which the remaining 7 traits loaded onto a single common factor. This model showed an improved fit, but was still not optimal (χ2 = 770.11, AIC = 798.11, CFI = 0.65, SRMR = 0.141), despite all the traits loading moderately to strongly onto the common factor, aside from SIB.

**Fig. 2:**
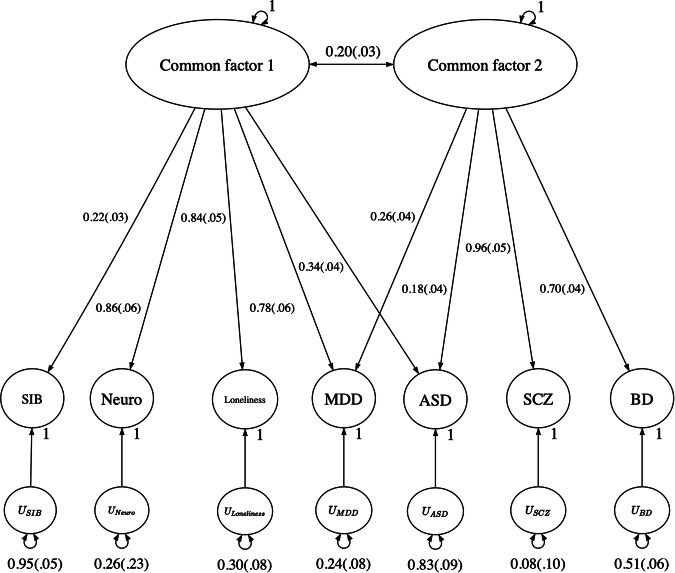
Genomic structural equation model of 11 traits loading onto 2 common latent genetic factors. The path diagram is displayed with the loadings of each trait with the standard errors in parentheses. *U* represents the residual variances after removing variance explained by the common factors.

Finally, we ran an exploratory factor analysis which suggested a 2-factor model, with personality loading onto one factor and psychotic traits (schizophrenia and BD) loading onto another, with MDD and ASD loading onto both (see Fig. 2). Confirmatory factor analysis demonstrated that this model is a good fit for the genetic data (χ2 = 87.06, AIC = 121.06, CFI = 0.97, SRMR = 0.067; see Supplementary Table 10 for full model fit statistics).

This model indicates 2 moderately correlated common genetic factors that distinctly influence personality and psychosis, with SIB loading less strongly than neuroticism and loneliness onto the personality factor. This finding may be due to SIB being a behavioral trait with the others being internalizing, despite being associated. Both personality and psychosis loading factors appear to influence MDD and ASD jointly. Overall, to some extent the genetic factors that increase SIB also increase neuroticism, loneliness, and depression, implicating behavior amongst the latter 3 traits previously found to be genetically correlated with each other and with general well-being [28, 29].

## Discussion

In this first study of the genetic factors that contribute to the behavior of social isolation (SIB), a meta-analysis of individual 4 behavioral trait GWAS of the discovery sample identified 17 genetic loci which predispose towards social isolation behavior. Some of these were in genes previously associated with psychiatric and neurological disorders, as well as neurotransmitter and brain function. However, most were not previously associated with other mental health, neurodevelopmental, or personality traits. Polygenic risk scores (PRS) derived from the GWAS were associated, in an independent developmental sample (ALSPAC), with the friendship scores at age 18 and there was strong evidence supporting shared genetic etiology between SIB and major psychiatric disorders, personality traits, and educational attainment, based on genetic correlations.

The PRS generated in ALSPAC were associated with friendship scores at age 18 but not at age 12. These results suggest that the SIB GWAS is a valid indicator of social-related traits, with higher PRS associated with lower friendship scores and outcomes. The PRS association with scores at age 18, as opposed to age 12, might indicate that genetically influenced personal social behavior does not necessarily manifest until later in adolescence. This finding could be due to confounding by gene-environment correlation [30]. At younger ages, children may have less control over their own social environments and interactions than at age 18, as their parents would likely select their environments for them, in which case behavior would be less strongly influenced by their own genetic predispositions. A similar effect is observed in intelligence genetics, in which heritability increases over time [31]. It is considered that genetic predisposition leads to active and passive correlations with school selection or teacher attention for example, creating a “snowball” effect in which those genetic influences are amplified over time. It is possible that similar effects are at play with behavioral genetics, in which SIB genetic predisposition leads to development, or lack thereof, of social skills and sociability, modulating real life social isolation over time.

SIB was found to be genetically correlated with schizophrenia, as well as with ASD and MDD. This pattern of results suggest that SIB is a feature that cuts across multiple psychiatric disorders and mental health generally. It is well known that social isolation is linked to poorer mental health [32], but here it is shown that there is a genetic association which indicates that SIB may form part of the etiological basis of these disorders. MR did not reveal evidence of causality of SIB on psychiatric disorders, but the specific method required for complex behavioral traits has limited power to detect such causality effects. Genomic SEM revealed a shared latent genetic component for SIB, MDD, ASD, loneliness, and neuroticism, and this component was also found to be moderately correlated with a shared genetic component which contributes to psychosis. Further studies with psychiatric populations will be required to test this hypothesis, but considering that social engagement is a modifiable intervention target [33], identifying those with a genetic predisposition towards SIB may be a useful strategy in mitigating mental health issues.

The current study demonstrated a heritable genetic component to SIB by utilizing a large sample size and detailed phenotype information in the discovery sample, allowing the development comprehensive and valid SIB trait. This was confirmed by the PRS generated from the discovery GWAS being validated in an independent sample, and several genome-wide significant SNPs were found associated with SIB. However, LD score correlation only estimated 4% heritability for SIB and PRS were only able to explain 0.6% of the variance in friendship scores in ALSPAC replication sample. The SNP-heritability is likely to be a lower bound estimate, as this only takes into account the common SNPs genotyped and not rare variants or de novo mutations [34]. Further, despite having up to 450,000 individuals available for the discovery GWAS, the most powerful GWAS such as educational attainment are becoming increasingly predictive with approximately 3 million participants [35]. Thus, increasing sample size will allow the detection of more SNPs that contribute to SIB behavior and increase both heritability estimates and the predictive power of PRS. In ALSPAC the target sample also had relatively few participants at age 18 (*n* = 2909) compared to age 12 (*n* = 4934), which likely contributed to lower bound variance explained.

Despite the likelihood that our results represent a lower bound estimate, the low heritability of SIB indicates that environmental factors also play a substantial role. Twin studies have shown that social isolation (as measured by proxy as low social support) has a 60% contribution of non-shared environmental factors [18]. This suggests that although genetics may drive social behavior and self-selection of isolating environments to some extent, sociodemographic factors, life events and peer influence may have a larger overall effect on the motivators of SIB. The heritable component of perceived social isolation or loneliness has been suggested as an adaptive mechanism that motivates connection and incentivizes group integration in the wake of the environmental risk of social isolation [36]. Our study suggests that genetic, as well as environmental factors play a role in the act of isolating the self, perhaps indicating adaptive benefits e.g., mitigating disease risk, competition, and risk of injury [37].

In order to further investigate how the genetic component of SIB manifests in behavior and the development of psychiatric disorders, further studies will be required establishing whether or not SIB PRS can predict case control status for disorders such as schizophrenia, MDD and ASD. If so, it will be necessary to consider which specific behaviors are influenced by genetics, and how they manifest in the development and diagnosis of psychiatric disorders. By targeting behavior, our present study has laid the foundation for identifying a possible target for intervention that can be addressed in real world scenarios. However, the relatively small effect sizes of individual SNPs and the resulting low predictive power of PRS indicate further investigation.

## Methods

### Study cohorts

#### Discovery sample

The UK Biobank (UKB) is a detailed prospective study with over 502,650 participants aged 40–69 years when recruited in 2006–2010 and includes both genetic and phenotypic data on complex traits [38]. The recruitment process was coordinated around 22 centers in the UK (between 2007 and 2010). Individuals within traveling distance of these centers were identified using NHS patient registers (response rate = 5.47%). Invitations were sent using a stratified approach to ensure demographic parameters were in concordance with the general population. All participants provided written informed consent and the current study was ethically approved by the UK Biobank Ethics and Governance Council (REC reference 11/NW/0382; UK Biobank application reference 18177).

#### Genetic data

Blood samples from 488,366 UK Biobank participants were genotyped using the UK BiLEVE array or the UK Biobank axiom array. Further details on the genotyping and quality control (QC) can be found on the UK Biobank website (http://www.ukbiobank.ac.uk/scientists-3/genetic-data/). In the current study, SNPs were removed if they had missingness <0.02 and a minor allele frequency (MAF) < 0.01. Exclusions based on heterozygosity and missingness were implemented according to UK Biobank recommendations (http://biobank.ctsu.ox.ac.uk/showcase/label.cgi?id=100314). Samples were removed if they were discordant for sex. SNPs deviating from Hardy-Weinberg equilibrium (HWE) were removed at a threshold of *P* < 1 × 10^−8^. Genotype data was imputed according to standard UK Biobank procedure, on 487,442 samples [39], excluding variants with an MAF < 0.01 and an imputation quality score < 0.3. After basic QC procedures and exclusions, 487,409 samples with phenotype data remained for genetic analysis. As genetic variants and polygenic risk scores (PRS) have low generalizability between ancestries, 4-means clustering on the first two principal components was performed, retaining 449,609 individuals from the largest cluster corresponding to European ancestry.

#### Phenotype data

##### Social isolation

To derive a comprehensive measure of social isolation behavior (SIB), we ran a data-driven principal component analyses (using Promax rotation) on all available self-reported answers to questions that (1) directly probed for the quantity or quality of social engagement, (2) were available for at least 90% of study participants, and (3) did not include internalizing, perceptions, or feelings related to sociability e.g. “Friendship satisfaction”. Based on these criteria, we included data on the following 3 items, that all loaded on a single factor: “Frequency of family/friend visits”, “Being able to confide in others”, and “Number of social activities a week”. The items “Frequency of family/friend visits” and “Being able to confide with others” were both rated on a seven-point Likert scale (i.e. ‘Almost daily’, ‘2–4 times a week’, ‘about once a week’, ‘about once a month’, ‘once every few months’, ‘never or almost never’, and ‘no friends/ family outside of household’). The items “Frequency of family/friend visits” and “Being able to confide with others” were considered continuously and recoded so that higher values corresponded to greater social isolation. Answer options for the item “Number of a/social activities a week” included attending a sports club, pub, social club, religious group, adult educational classes, or other group activities and were summed to represent the ‘total number of social activities a week’, also considered continuously.

To complement the answers to the self-report, sociodemographic information about the number of people in the household was added as an additional proxy of social contact, as representing a behavioral decision to isolate. This “Number in household” item was dichotomized as a binary trait representing living alone, with 0 others in household coded as ‘1’ for SIB and any greater number in household as ‘0’. As such, higher scores for each of the 4 traits corresponded to higher SIB. See supplementary material for full phenotype and coding details, and https://biobank.ndph.ox.ac.uk/showcase/ for further information. For all items, individuals with missing data, or who preferred not to answer were excluded (maximum N = 18949). Participants who were wheelchair users (*N* = 426) and/or morbidly obese (BMI > 40; *N* = 9689) were also excluded from the analysis, as these factors may arguably hamper the level of social activity but are unrelated to genetic or psychiatric vulnerability to SIB [40, 41].

#### Validation cohort

##### ALSPAC cohort

The Avon Longitudinal Study of Parents and Children (ALSPAC) is a prospective birth cohort which recruited pregnant women with expected delivery dates between April 1991 and December 1992 from Bristol UK. 14,541 pregnant women were initially enrolled with 14,062 children born and 13,988 alive at 1 year of age. Detailed information on health and development of children and their parents were collected from regular clinic visits and completion of questionnaires. Please note that the study website contains details of all the data that is available through a fully searchable data dictionary and variable search tool” and reference the following webpage: http://www.bristol.ac.uk/alspac/researchers/our-data/. A detailed description of the cohort has been previously published [42, 43]. Ethical approval for the study was obtained from the ALSPAC Ethics and Law Committee and the Local Research Ethics Committees.

#### Genotype data

11,343 participants in ALSPAC have genotype data available, genotyped using the Illumina HumanHap550 quad chip genotyping platforms, and standard quality control (QC) procedures applied. Individuals with non-European ancestry were removed to minimize bias due to ancestral population stratification. SNPs with a MAF of <0.01, a call rate of <0.95 or evidence for violations of Hardy–Weinberg equilibrium (*P* < 5 × 10^−07^) were removed. Data was imputed using standard ALSPAC procedure using the HapMap 2 reference panel, keeping SNPs with MAF > 0.02 and an INFO score >0.9. After these quality control measures, 9115 individuals and 4,731,235 SNPs remained in the analysis. Full quality control procedures can be found at: https://alspac.github.io/omics_documentation/alspac_omics_data_catalogue.html

#### Phenotype data

To test the validity of the SIB construct, 2 friendship scores were derived from 5 questions from clinical questionnaires based on questions from the Cambridge Hormones and Moods Project Friendship Questionnaire [44], completed by the parents of offspring at ages 12 and 18 respectively e.g. “*Teenager is happy with number of friends”*. Each question consisted of 4–6 categorical responses, corresponding to a 4–6 point scale e.g. “1 = Very happy, 2 = Quite happy, 3 = Quite unhappy, 4 = Unhappy, 5 = No friends”. Responses were summed to create a continuous scale, with higher scores corresponding to lower friendship quality and greater SIB. 4, 934 of the cohort had the phenotype information at age 12, and 2909 at age 18. See supplementary Table 2 for full details on questions.

##### GWAS summary statistics

To test for genetic correlations between SIB and associated psychiatric disorders using LD score correlation, the SIB GWAS based on UK Biobank data was used along 10 base genome-wide association summary statistics for schizophrenia, depression (MDD), autism spectrum disorder (ASD), anorexia nervosa (AN), bipolar disorder (BD), anxiety, extraversion, neuroticism, loneliness, and educational attainment. These were the Psychiatric Genomics Consortium Wave 3 (PGC3) schizophrenia GWAS [45], the 2019 PGC MDD Working Group GWAS [46], and the 2017 PGC ASD Working Group GWAS [47], the 2019 Eating Disorders Working Group of the Psychiatric Genomics Consortium anorexia GWAS [48], the BD Working group of the Psychiatric Genomics Consortium GWAS [49], the 2019 iPSYCH anxiety GWAS [50] the Genetics of Personality-2 (GPC-2) extraversion and neuroticism GWAS [51, 52], a 2019 GWAS meta-analysis of loneliness [53], and the 2018 Social Science Genetic Association Consortium (SSGAC) GWAS of educational attainment [54].

### Statistical analyses

#### GWAS analysis

Association testing of autosomal SNPs was carried out on each of the 4 SIB traits (“*Frequency of family/friend visits*”, “*Being able to confide in others*”, “*Number of social activities a week*”, and “*Number in household”*) using BOLT Bayesian linear mixed models (BOLT-LMM) [55] to account for relatedness and cryptic population stratification, while increasing power and controlling for false positives. Age, sex, batch, and center were included as covariates, as well as education, income, and Townsend deprivation index (TDI) to account for socio-economic status (SES). The top 15 principal components (PCs) were also included to control for main population stratification. Multi-Trait Analysis of GWAS (MTAG) [56] was used to meta-analyze the individual “*Frequency of family/friend visits*”, “*Being able to confide in others*”, “*Number of social activities a week*”, and “*Number in household”* outcomes to form a single, composite SIB GWAS. This score is achieved by leveraging power across correlated GWAS estimates in overlapping samples. Finally, multitrait-based conditional and joint analysis (mtCOJO) [57] was used to adjust the SIB GWAS summary statistics for the effects of psychiatric disorders, specifically schizophrenia, major depressive disorder (MDD), and autism spectrum disorder (ASD), using European ancestry GWAS summary statistics for each. These are the psychiatric disorders which are commonly considered to lead to increased risk of social withdrawal and isolation [2–4, 7, 8] and were conditioned on to remove potential downstream effects of psychiatric disorders. SNPs were selected as instruments at 5 × 10^−05^, clumped 1MB apart or with LD *r*^*2*^ < 0.2 based on the 1000 Genomes Project Phase 3 reference panel for independence. mtCOJO uses these SNPs Generalized Summary-data-based Mendelian Randomization (GSMR) to estimate the effect of the exposures (psychiatric disorders) on the outcome (SIB), producing conditioned effect sizes and *p*-values. Statistically significant independent signals were identified using 1MB clumping and a genome-wide significance threshold of *P* < 5 × 10^−08^.

#### Polygenic risk score analysis

Polygenic risk scores (PRS) were generated in ALSPAC using PRSice-2 [58], using the discovery SIB GWAS to sum and weight risk alleles for individuals in each cohort. SIB GWAS results were pruned for linkage disequilibrium (LD) using the *p*-value informed clumping method in PLINK (-clump-p1 1 - clump-p2 1 -clump-r2 0.1 -clump-kb 250). This method preferentially retains SNPs with the strongest evidence of association and removes SNPs in LD (r2 > 0.1) that show weaker evidence of association within 250Kb windows, based on LD structure from the HRC reference panel. Subsets of SNPs were selected from the results at 13 increasingly liberal P value thresholds (ranging from p < 5 × 10^−08,^ to *p* < 0.5). Risk alleles were included and tested to predict outcomes at 13 different significance thresholds, allowing the utilization of the most predictive PRS and threshold. These PRS were tested for associations with the friendship scores in ALSPAC, using linear regression models and including age, sex and 10 PCs as covariates. To account for the multiple testing of 13 PRS thresholds and 2 friendship scores, a Bonferroni correct significance threshold of *P* < 0.002 was used.

#### LD score correlation

Genetic correlations and heritability estimates were conducted using LD score correlation [59], to investigate associations between SIB and schizophrenia, MDD, ASD, AN, BD, anxiety, extraversion, neuroticism, loneliness, and educational attainment, using GWAS summary statistics from the SIB GWAS conducted in the UK Biobank and each psychiatric disorder or trait from the PGC, GPC, iPSYCH, or SSGAC. LD score correlation provides an accurate estimation of the genetic correlation between two traits by separating genuine polygenic effects from confounders by regressing test statistics against LD scores (correlation between genomic sites, and SNPs tagging causal variants), as SNPs with test statistics describing true associations are positively correlated with LD scores, and the intercept estimates confounding accurately.

#### Mendelian randomization

To test for causality between SIB and psychiatric outcomes, bi-directional Mendelian Randomization was conducted using the r-package TwoSampleMR (https://github.com/MRCIEU/TwoSampleMR) [60]. Instrumental variables for the exposures (both SIB and the significant genetically correlated schizophrenia, MDD, ASD, extraversion, loneliness, and educational attainment) were extracted at genome-wide significance and at *p* < 5 × 10^−06^ after strict LD clumping at 10,000 kb windows and LD *r*^*2*^ < 0.001 to ensure instruments were independent. Exposure and outcomes were harmonized and MR-Egger was used in the primary analyses to account for horizontal pleiotropy. The inverse variance weighted (IVW) was also used as a less conservative, more powerful approach as its weighting combines instrumental variable estimates more efficiently, but it may not be valid for these traits as it assumes no horizontal pleiotropy is present. To account for multiple testing, a Bonferroni corrected p-value threshold of *P* < 0.004 was used to ascertain significance.

#### Genomic SEM

To investigate the genetic architecture of the traits investigated along with SIB, we performed exploratory and confirmatory factor analysis using the r-package Genomic Structural Equation Modelling (Genomic SEM; https://github.com/GenomicSEM/GenomicSEM) [61]. Genomic SEM uses multivariable LD score correlation to fit models using genetic and sampling covariance matrices. A single common genomic factor is derived with loadings for each trait which represent shared genetic contribution. Exploratory factor analysis was then performed of the genetic covariance matrix to determine loadings across multiple factors. Confirmatory factor analysis was then utilized to compare and test model fits.

## Supplementary information

Supplementary material

## Data Availability

UK Biobank data are available through a procedure described at http://www.ukbiobank.ac.uk/using-the-resource/. ALSPAC data access is through a system of managed open access. The steps below highlight how to apply for access to the data included in this paper and all other ALSPAC data. 1. Please read the ALSPAC access policy (http://www.bristol.ac.uk/media-library/sites/alspac/documents/researchers/data-access/ALSPAC_Access_Policy.pdf) which describes the process of accessing the data and biological samples in detail, and outlines the costs associated with doing so. 2. You may also find it useful to browse our fully searchable research proposals database (https://proposals.epi.bristol.ac.uk/), which lists all research projects that have been approved since April 2011. 3. Please submit your research proposal (https://proposals.epi.bristol.ac.uk/) for consideration by the ALSPAC Executive Committee using the online process. You will receive a response within 10 working days to advise you whether your proposal has been approved. If you have any questions about accessing data, please email: alspac-data@bristol.ac.uk. Schizophrenia, Autism spectrum disorder, major depressive disorder, bipolar disorder, and anorexia nervosa GWAS summary statistics are publicly available from the PGC (https://www.med.unc.edu/pgc/download-results/). Anxiety GWAS summary statistics are available from iPSYCH (https://ipsych.dk/en/research/downloads). Extraversion and Neuroticism GWAS summary statistics are publicly available from the GPC (https://tweelingenregister.vu.nl/gpc). Loneliness GWAS summary statistics are available at the following link: https://t.co/ARgS84uwKl. Educational attainment GWAS summary statistics are available from the SSGAC data portal (https://thessgac.com/).

## Code availability

Software code for *PRSice-2* is available at https://www.prsice.info/. All other code used is available upon request.
