# Supplementary material for: Polygenic risk of social isolation behavior and its influence on psychopathology and personality
Source: Mol Psychiatry. 2024 May 30;29(11):3599–606. doi: 10.1038/s41380-024-02617-2 (PMC11541194; doi:10.1038/s41380-024-02617-2)
Supplement: Supplementary file 1 — Supplementary material [file 41380_2024_2617_MOESM1_ESM.docx]

**Supplementary material**

**Supplementary Table 1. Social isolation phenotype details**

| Phenotype name | Touchscreen question | Response coding |
| --- | --- | --- |
| Frequency of friend/family visits | "How often do you visit friends or family or have them visit you?" | \| 1 \| Almost daily \| \| --- \| --- \| \| 2 \| 2-4 times a week \| \| 3 \| About once a week \| \| 4 \| About once a month \| \| 5 \| Once every few months \| \| 6 \| Never or almost never \| \| 7 \| No friends/family outside household \| \| NA \| Do not know \| \| NA \| Prefer not to answer \| |
| Being able to confide in others | "How often are you able to confide in someone close to you?" | \| 0 \| Almost daily \| \| --- \| --- \| \| 1 \| 2-4 times a week \| \| 2 \| About once a week \| \| 3 \| About once a month \| \| 4 \| Once every few months \| \| 5 \| Never or almost never \| \| NA \| Do not know \| \| NA \| Prefer not to answer \| |
| Number of social activities a week | “Which of the following do you attend once a week or more often? (You can select more than one)” | \| 1 \| Sports club or gym \| \| --- \| --- \| \| 2 \| Pub or social club \| \| 3 \| Religious group \| \| 4 \| Adult education class \| \| 5 \| Other group activity \| \| 0 \| None of the above \| \| NA \| Prefer not to answer* \| \|  \|  \| |
| Number in household | Including yourself, how many people are living together in your household? (Include those who usually live in the house such as students living away from home during term, partners in the armed forces or professions such as pilots)" | \| 0 \| More than one (living with others) \| \| --- \| --- \| \| 1 \| One (living alone) \| |

***Coded as cumulative score based on number of social activities and recoded so fewer social activities correspond to a higher SIB score**

**Supplementary Table 2. Age 12 friendship score items from ALSPAC**

| Teenager is happy with number of friends | Higher number = greater unhappiness. Highest score of 5 = no friends |
| --- | --- |
| Frequency teenager sees friends out of school | Higher number = less frequency. Highest score of 6 = never |
| Teenager believes friends understand them | Higher number = less understanding. Highest score of 4 = not at all |
| Teenager talks about problems with friends | Higher number = less talking. Highest score of 4 = not at all |
| Degree to which teenager is happy with his/her friends overall | Higher number = greater unhappiness. Highest score of 4 = Unhappy |

***friend12_5q: Total score, with higher score = poorer friendship quality (based on the available items also at age 18; 5)**

**Supplementary Table 3. Age 18 friendship score items from ALSPAC**

| Young person is happy with the number of friends they have | Higher number = greater unhappiness. Highest score of 5 = no friends |
| --- | --- |
| Frequency young person meets with their friends outside college/work | Higher number = less frequency. Highest score of 6 = never |
| Young person thinks that their friends understand them | Higher number = less understanding. Highest score of 4 = not at all |
| Young person talks to any of their friends about their problems | Higher number = less talking. Highest score of 4 = not at all |
| How happy young person is overall with their friends | Higher number = greater unhappiness. Highest score of 4 = Unhappy |

***friend18_5q: Total score, with higher score = poorer friendship quality (based on all available items; 5)**

**Supplementary Table 4. Linkage disequilibrium (LD) Score regression showing genetic correlation between “Able to confide” and other social isolation behavior (SIB) traits**

| **Trait 1** | **Trait 2** | **rg** | **se** | **z** | **p** | **h2 obs** | **h2 obs se** | **h2 int** | **h2 int se** |
| --- | --- | --- | --- | --- | --- | --- | --- | --- | --- |
| Able to confide | Frequency family friends | 0.3525 | 0.03 | 11.748 | 7.23E-32 | 0.0357 | 0.0021 | 1.0274 | 0.0085 |
| Able to confide | Number in household | 0.4312 | 0.0431 | 9.9947 | 1.61E-23 | 0.0131 | 0.0013 | 1.0201 | 0.0073 |
| Able to confide | Social activities | 0.1711 | 0.0292 | 5.8578 | 4.69E-09 | 0.036 | 0.0019 | 1.0163 | 0.0084 |

**Supplementary Table 5. Social isolation behavior (SIB) genome-wide association study (GWAS) results**

| **CHR** | **F** | **SNP** | **BP** | **P** |
| --- | --- | --- | --- | --- |
| 20 | 1 | rs67777906 | 47557129 | 1.80E-15 |
| 8 | 1 | rs10099728 | 65260306 | 1.81E-11 |
| 3 | 1 | rs11711331 | 81819040 | 1.15E-10 |
| 8 | 1 | rs2721942 | 116637744 | 1.47E-10 |
| 19 | 1 | rs28567442 | 30930222 | 6.31E-10 |
| 6 | 1 | rs4524616 | 98445008 | 9.33E-10 |
| 6 | 1 | rs13208578 | 98572976 | 1.26E-09 |
| 19 | 1 | rs17614190 | 30951753 | 2.45E-09 |
| 3 | 1 | rs12497231 | 81519291 | 3.39E-09 |
| 1 | 1 | rs862994 | 159165336 | 3.68E-09 |
| 20 | 1 | rs6125539 | 47703810 | 4.72E-09 |
| 13 | 1 | rs17057528 | 60600310 | 8.82E-09 |
| 3 | 1 | rs1248860 | 85015779 | 9.51E-09 |
| 20 | 1 | rs6012544 | 47488807 | 1.85E-08 |
| 20 | 1 | rs2426154 | 48001995 | 2.26E-08 |
| 17 | 1 | rs62084697 | 66038556 | 4.56E-08 |
| 2 | 1 | rs11123811 | 100760172 | 4.77E-08 |

**Supplementary Table 6. Full results of social isolation behavior (SIB) polygenic risk scores (PRS) predicting friendship scores (age 12)**

| **Outcome** | **Exposure** | **PRS p threshold** | **Coefficient** | **SE** | **P** | **Lower CI** | **Upper CI** | **r2** | **N** |
| --- | --- | --- | --- | --- | --- | --- | --- | --- | --- |
| friend12_5q | std_score_S1_EurRel | 0.5 | 0.062 | 0.030 | 0.042 | 0.002 | 0.121 | 0.009 | 4934 |
| friend12_5q | std_score_S2_EurRel | 0.4 | 0.064 | 0.030 | 0.035 | 0.005 | 0.123 | 0.009 | 4934 |
| friend12_5q | std_score_S3_EurRel | 0.3 | 0.063 | 0.030 | 0.038 | 0.004 | 0.122 | 0.009 | 4934 |
| friend12_5q | std_score_S4_EurRel | 0.2 | 0.067 | 0.030 | 0.028 | 0.007 | 0.126 | 0.009 | 4934 |
| friend12_5q | std_score_S5_EurRel | 0.1 | 0.062 | 0.030 | 0.040 | 0.003 | 0.122 | 0.009 | 4934 |
| friend12_5q | std_score_S6_EurRel | 0.05 | 0.056 | 0.030 | 0.064 | -0.003 | 0.116 | 0.008 | 4934 |
| friend12_5q | std_score_S7_EurRel | 0.01 | 0.011 | 0.030 | 0.711 | -0.048 | 0.071 | 0.008 | 4934 |
| friend12_5q | std_score_S8_EurRel | 0.001 | 0.008 | 0.030 | 0.785 | -0.051 | 0.068 | 0.008 | 4934 |
| friend12_5q | std_score_S9_EurRel | 1.00E-04 | -0.028 | 0.030 | 0.351 | -0.088 | 0.031 | 0.008 | 4934 |
| friend12_5q | std_score_S10_EurRel | 1.00E-05 | 0.018 | 0.031 | 0.552 | -0.042 | 0.079 | 0.008 | 4934 |
| friend12_5q | std_score_S11_EurRel | 1.00E-06 | 0.023 | 0.031 | 0.448 | -0.037 | 0.084 | 0.008 | 4934 |
| friend12_5q | std_score_S12_EurRel | 1.00E-07 | -0.002 | 0.031 | 0.956 | -0.062 | 0.059 | 0.008 | 4934 |
| friend12_5q | std_score_S13_EurRel | 5.00E-08 | 0.002 | 0.031 | 0.960 | -0.059 | 0.062 | 0.008 | 4934 |

**Supplementary Table 7. Full results of social isolation behavior (SIB) polygenic risk scores (PRS) predicting friendship scores (age 18)**

| **Outcome** | **Exposure** | **PRS p threshold** | **Coefficient** | **SE** | **P** | **Lower CI** | **Upper CI** | **r2** | **N** |
| --- | --- | --- | --- | --- | --- | --- | --- | --- | --- |
| friend18_5q | std_score_S1_EurRel | 0.5 | 0.144 | 0.049 | 0.003 | 0.048 | 0.239 | 0.005 | 2909 |
| friend18_5q | std_score_S2_EurRel | 0.4 | 0.139 | 0.049 | 0.004 | 0.043 | 0.234 | 0.005 | 2909 |
| friend18_5q | std_score_S3_EurRel | 0.3 | 0.142 | 0.049 | 0.003 | 0.047 | 0.238 | 0.005 | 2909 |
| friend18_5q | std_score_S4_EurRel | 0.2 | 0.150 | 0.049 | 0.002 | 0.054 | 0.245 | 0.005 | 2909 |
| friend18_5q | std_score_S5_EurRel | 0.1 | 0.169 | 0.049 | 0.001 | 0.073 | 0.265 | 0.006 | 2909 |
| friend18_5q | std_score_S6_EurRel | 0.05 | 0.158 | 0.049 | 0.001 | 0.062 | 0.253 | 0.005 | 2909 |
| friend18_5q | std_score_S7_EurRel | 0.01 | 0.099 | 0.049 | 0.043 | 0.003 | 0.196 | 0.003 | 2909 |
| friend18_5q | std_score_S8_EurRel | 0.001 | 0.099 | 0.050 | 0.046 | 0.002 | 0.196 | 0.003 | 2909 |
| friend18_5q | std_score_S9_EurRel | 1.00E-04 | 0.077 | 0.050 | 0.125 | -0.021 | 0.175 | 0.003 | 2909 |
| friend18_5q | std_score_S10_EurRel | 1.00E-05 | 0.073 | 0.051 | 0.151 | -0.026 | 0.172 | 0.002 | 2909 |
| friend18_5q | std_score_S11_EurRel | 1.00E-06 | 0.038 | 0.051 | 0.456 | -0.061 | 0.137 | 0.002 | 2909 |
| friend18_5q | std_score_S12_EurRel | 1.00E-07 | 0.059 | 0.050 | 0.241 | -0.040 | 0.157 | 0.002 | 2909 |
| friend18_5q | std_score_S13_EurRel | 5.00E-08 | 0.060 | 0.050 | 0.234 | -0.039 | 0.159 | 0.002 | 2909 |

**Supplementary Table 8. Full results of linkage disequilibrium (LD) Score regression genetic correlations.**

| **Trait 1** | **Trait 2** | **rg** | **se** | **z** | **p** |
| --- | --- | --- | --- | --- | --- |
| Social isolation behavior | Schizophrenia | 0.102 | 0.03 | 3.67 | 0.0002 |
| Social isolation behavior | Major depressive disorder | 0.093 | 0.04 | 2.61 | 0.009 |
| Social isolation behavior | Autism spectrum disorder | 0.228 | 0.05 | 4.73 | 2.20E-06 |
| Social isolation behavior | Anorexia Nervosa | -0.073 | 0.04 | -1.79 | 0.073 |
| Social isolation behavior | Bipolar disorder | -0.018 | 0.04 | -0.51 | 0.61 |
| Social isolation behavior | Anxiety | -0.058 | 0.09 | -0.67 | 0.5 |
| Social isolation behavior | Extraversion | -0.438 | 0.06 | -6.77 | 1.3E-11 |
| Social isolation behavior | Neuroticism | -0.001 | 0.07 | -0.01 | 0.99 |
| Social isolation behavior | Loneliness | 0.289 | 0.03 | 9.24 | 2.38E-20 |
| Social isolation behavior | Educational attainment | 0.129 | 0.02 | 5.19 | 2.10E-07 |

**Supplementary Table 9. Full results of Mendelian randomization.**

| **Exposure** | **Outcome** | **Method** | **N SNPs** | **B** | **SE** | **P** |
| --- | --- | --- | --- | --- | --- | --- |
| SIB | ASD | MR Egger | 65 | -69.360 | 52.57 | 0.192 |
| ASD | SIB | MR Egger | 23 | 0.020 | 0.01 | 0.025 |
| SIB | ASD | Inverse variance weighted | 65 | 0.391 | 9.44 | 0.967 |
| ASD | SIB | Inverse variance weighted | 23 | 0.000 | 0.00 | 0.740 |
| SIB | SCZ | MR Egger | 65 | 1.008 | 54.72 | 0.985 |
| SCZ | SIB | MR Egger | 313 | 0.004 | 0.00 | 0.225 |
| SIB | SCZ | Inverse variance weighted | 65 | 4.183 | 9.49 | 0.659 |
| SCZ | SIB | Inverse variance weighted | 313 | 0.000 | 0.00 | 0.706 |
| SIB | MDD | MR Egger | 65 | -70.943 | 52.86 | 0.184 |
| MDD | SIB | MR Egger | 34 | 0.000 | 0.01 | 0.974 |
| SIB | MDD | Inverse variance weighted | 65 | 0.782 | 9.47 | 0.934 |
| MDD | SIB | Inverse variance weighted | 34 | 0.000 | 0.00 | 0.339 |
| SIB | Edu | MR Egger | 65 | 0.361 | 0.28 | 0.196 |
| Edu | SIB | MR Egger | 440 | 0.085 | 0.05 | 0.120 |
| SIB | Edu | Inverse variance weighted | 65 | 0.080 | 0.05 | 0.102 |
| Edu | SIB | Inverse variance weighted | 440 | 0.087 | 0.01 | 0.000 |
| SIB | Extra | MR Egger | 65 | -0.029 | 0.41 | 0.945 |
| Extra | SIB | MR Egger | 8 | 0.133 | 0.14 | 0.386 |
| SIB | Extra | Inverse variance weighted | 65 | -0.187 | 0.07 | 0.009 |
| Extra | SIB | Inverse variance weighted | 8 | 0.071 | 0.03 | 0.038 |
| SIB | Loneliness | MR Egger | 63 | 0.789 | 0.32 | 0.016 |
| Loneliness | SIB | MR Egger | 53 | 0.429 | 0.16 | 0.010 |
| SIB | Loneliness | Inverse variance weighted | 63 | 0.312 | 0.06 | 0.000 |
| Loneliness | SIB | Inverse variance weighted | 53 | 0.270 | 0.03 | 0.000 |

*SIB = social isolation behavior; ASD = autism spectrum disorder; SCZ = schizophrenia; MDD = major depressive disorder; Edu = educational attainment; Extra = extraversion

N SNPS = number of SNPs in exposure; B = Beta; SE = standard errors; P = *p*-value

**Supplementary Table 10. Model fit statistics for each of the 3 described Genomic structural equation modelling (SEM) models**

| **Model** | **χ2** | **df** | **p-value** | **AIC** | **CFI** | **SRMR** |
| --- | --- | --- | --- | --- | --- | --- |
| Common factor model with 11 traits | 1756.37 | 44 | 0.00E+00 | 1800.37 | 0.54 | 0.13 |
| Common factor model with 7 traits | 770.11 | 14 | 2.73E-155 | 798.11 | 0.65 | 0.14 |
| 2-factor model with 7 traits | 87.06 | 11 | 6.28E-14 | 121.06 | 0.97 | 0.07 |

**χ2** = chi-squared test statistic; **df** = degrees of freedom; **AIC** = Akaike’s Information criterion; **CFI** = Comparitive Fit Index; **SRMR** = standardized root mean squared residual. Good fit is indicated by CFI > 0.90 and SRMR < 0.08.
